# Radiological Underestimation of Tumor Size as a Relevant Risk Factor for Positive Margin Rate in Breast-Conserving Therapy of Pure Ductal Carcinoma In Situ (DCIS)

**DOI:** 10.3390/cancers14102367

**Published:** 2022-05-11

**Authors:** Gesche Schultek, Bernd Gerber, Toralf Reimer, Johannes Stubert, Steffi Hartmann, Annett Martin, Angrit Stachs

**Affiliations:** Department of Obstetrics and Gynecology, University of Rostock, 18059 Rostock, Germany; bernd.gerber@kliniksued-rostock.de (B.G.); toralf.reimer@kliniksued-rostock.de (T.R.); johannes.stubert@kliniksued-rostock.de (J.S.); steffi.hartmann@kliniksued-rostock.de (S.H.); annett.martin@kliniksued-rostock.de (A.M.); angrit.stachs@uni-rostock.de (A.S.)

**Keywords:** ductal carcinoma in situ, breast-conserving surgery, positive margin rate, radiological underestimation

## Abstract

**Simple Summary:**

Negative margins are the most important prognostic factor in breast-conserving therapy (BCT) of ductal carcinoma in situ (DCIS). The impact of radiological underestimation ≥10 mm (defined as mammographic minus histological tumor size in millimeters) has not been further examined. The purpose was to verify the radiological underestimation of DCIS size as a risk factor for positive margins. A pooled analysis of two trials was performed. Inclusion criteria were patients receiving BCT in DCIS. The results show a clinically relevant radiological underestimation in 37% of patients. Radiological underestimation is an independent risk factor for positive margins in BCT of DCIS with microcalcifications. Furthermore, the influencing factors of radiological underestimation were analysed. In multivariate logistic regression, only a mammographic tumor size ≤20 mm was an independent risk factor associated with radiological underestimation. When planning and executing BCT, it has to be considered that a relevant radiological underestimation is significantly higher in mammographic DCIS sizes ≤20 mm.

**Abstract:**

Background: Radiological underestimation of the actual tumor size is a relevant problem in reaching negative margins in ductal carcinoma in situ (DCIS) associated with microcalcifications in breast-conserving therapy (BCT). The aim of this study is to evaluate whether the radiological underestimation of tumor size has an influence on the histopathological margin status. Methods: Patients who underwent BCT with preoperatively diagnosed pure DCIS were included (pooled analysis of two trials). Multiple factors were analysed regarding radiological underestimation ≥10 mm. Radiological underestimation was defined as mammographic minus histological tumor size in mm. Results: Positive margins occurred in 75 of 189 patients. Radiological underestimation ≥10 mm was an independent influencing factor (OR 5.80; 95%CI 2.55–13.17; *p* < 0.001). A radiological underestimation was seen in 70 patients. The following parameters were statistically significant associated with underestimation: pleomorphic microcalcifications (OR 3.77; 95%CI 1.27–11.18), clustered distribution patterns (OR 4.26; 95%CI 2.25–8.07), and mammographic tumor sizes ≤20 mm (OR 7.47; 95%CI 3.49–15.99). Only a mammographic tumor size ≤20 mm was an independent risk factor (OR 6.49; 95%CI 2.30–18.26; *p* < 0.001). Grading, estrogen receptor status, and comedo necrosis did not influence the size estimation. Conclusion: Radiological underestimation is an independent risk factor for positive margins in BCT of DCIS associated with microcalcifications predominantly occurring in mammographic small tumors.

## 1. Introduction

Sixty to ninety percent of noninvasive ductal carcinoma in situ (DCIS) is associated with microcalcifications [1]. Since the introduction of widely spread screening programs, DCIS has been frequently diagnosed. Thus, 20–25% of all breast cancer diagnoses are DCIS [2,3,4,5]. Untreated DCIS may progress to invasive breast cancer (IBC) in up to 30–50% of patients over a period of 10 years [6,7,8]. The disease-specific mortality in DCIS is low [9], but the local recurrence rate is, on average 30% [10,11], and up to 50% of recurrences are invasive [12,13]. The standard therapy includes surgery, and in high-risk cases, radiotherapy as well as endocrine therapy. Even though wire-guided breast-conserving surgery (BCS) is the most commonly used surgery in both invasive breast cancer and DCIS, positive margins are more often seen in DCIS than in IBC [5,14,15,16]. Therefore, re-operations are four times as likely for cases of pure DCIS versus those containing an IBC component [17,18,19,20,21]. Other studies revealed re-operation rates between 14 and 78% [2,22,23,24,25,26].

Recent studies involving DCIS investigated the issue of local recurrences and the definition of negative margins (negative margin of 2 mm in only DCIS, in combination with invasive disease and no ink on tumor). Positive margins increase the risk of in-breast recurrence (IBR) [27,28]. The margin status remains an important risk factor in local recurrence [29]. Achieving negative margins in the initial surgery of a DCIS is more difficult compared to invasive breast cancer. Positive margins depend on many factors; for example, comedo necrosis, radiological margins <10 mm [30,31], negative progesterone receptor (PR), tumor grade, and larger DCIS size [20,31,32]. Intraoperative margin assessment is challenging in pure DCIS. However, evidence concerning the prevention of positive margins is low. A recent review did not support the use of intraoperative specimen radiography for the reduction in positive margins [15]. One of the limitations could be radiological over- and underestimations of the actual DCIS size, which could influence the margin status.

The primary aim of this study was to evaluate whether the radiological underestimation of tumor size has an influence on the histopathological margin status. The secondary aim was to analyze preoperatively known variables that are potentially associated with radiological underestimation.

## 2. Materials and Methods

Two trials were part of the pooled analysis: a retrospective trial from 2011 (PMID: 27017245) and a current prospective validation study concerning specimen radiography (DRKS00011527). The study was approved by the local ethics committee of the University of Rostock, Germany. Patients with the diagnosis of DCIS (preoperatively diagnosed with a core needle biopsy or vacuum suction biopsy) associated with microcalcifications who underwent BCS at the Breast Unit of the Clinic of Obstetrics and Gynecology at the University of Rostock were included. Preoperative mammograms had to be available in DICOM format. Exclusion criteria were patients with a planned mastectomy, prior breast surgery, as well as DCIS without microcalcification. Radiological underestimation was defined as the mammographic minus histological tumor size in mm. We considered a size difference of ≥10 mm as clinically relevant.

A preoperative wire localization (Somatex Medical Technologies GmbH, Berlin, Germany) of the suspicious microcalcifications under mammographic guidance was performed by an experienced breast radiologist (A.S.). Adapted on the size of the area of microcalcifications, flanking wire localization was performed if necessary. The surgery was executed by experienced breast surgeons (B.G., T.R., J.S., A.S., S.H., and A.M.). After the BCS, the orientation was given by sutures on the specimen or by using a radiopaque tissue transfer and X-ray system (KlinitrayRM, Klinika medical GmbH, Usingen, Germany).

Intraoperative specimen radiography was used to evaluate the margins. Radiologically positive margins resolved in an intraoperative re-excision. After surgery, the histological specimen was worked up and underwent histological examination. The largest histopathological tumor diameter was determined as a reference standard. Histopathological negative margins were defined as ≥2 mm, or if skin and/or fascia were on the margin.

The collected data included multiple histological features (tumor size, comedo necrosis, estrogen receptor status, and margins) and radiological information (mammographic tumor size, multifocality, and radiological margins). All mammograms and specimen radiographs were blind-reviewed by an experienced breast radiologist (A.S.) for mammographic tumor size, distribution pattern, and morphology of microcalcifications. It was also documented whether or not an intraoperative re-excision had been performed.

For statistical analysis, SPSS 27.0 software (IBM, Armonk, New York, NY, USA) was used. Descriptive statistics for nominal (scaled) and quantitative (scaled) variables were computed (percentages, frequencies, mean, standard deviation, median, minimum, and maximum). To test for significant differences, the chi-square test and Fisher’s exact test, as well as the t-test and Mann–Whitney U test, were used whenever appropriate. All *p* values resulted from two-sided statistical tests, and values of *p* < 0.05 were regarded to be statistically significant. To describe risk factors for positive margins after the initial BCS, univariate binary and multivariable logistic regression were performed, and crude and adjusted odds ratios (OR) with a 95% confidence interval (CI), as well as *p* values, were calculated.

## 3. Results

### 3.1. Risk Factors Associated with Positive Margins

We analyzed 189 patients with pure DCIS. The median age was 59.7 years (range: 34–84 years). Histologically positive margins were found in 75 (39.7%) patients. The median tumor size differed significantly in patients with positive margins compared to those with negative margins (31.0 mm in patients with positive margins vs. 17.0 mm in patients with negative margins, *p* < 0.001). There was also a difference in the median mammographic tumor size (22.0 vs. 13.0 mm, *p* = 0.011) and the frequency of radiological margins <5 mm in specimen radiography (72% vs. 50%, *p* = 0.004) between patients with histologically involved or not-involved margins. There was a significantly higher percentage of radiological underestimation ≥10 mm in patients with positive margins compared to those with free margins (49.3% vs. 28.9%, *p* = 0.006). Tumor biological factors such as negative estrogen and progesterone receptor status were associated with positive margins, whereas the grade of differentiation was not (Table 1).

In a univariate analysis, a histological DCIS size >25 mm was associated with a sevenfold increased risk for positive margins (OR 7.36; 95%CI 3.82–14.2). Patients with a mammographic DCIS size >20 mm, a negative estrogen and progesterone receptor, a radiological margin width <5 mm, and mammographic underestimation ≥10 mm were more likely to have positive margins after the initial BCS. For calculation of adjusted odds ratios, histological tumor size was excluded, since this factor was too strong for the assessment of other variables. In multivariable regression analysis, mammographic underestimation was associated with a nearly sixfold increased risk for positive margins (adj. OR 5.81; 95%CI2.39–14.12). Further independent risk factors were specimen sizes <50 mm, mammographic tumor sizes >20 mm, and radiological margins <5 mm (Table 2).

### 3.2. Mammographic Size Estimation

**Figure 1:**
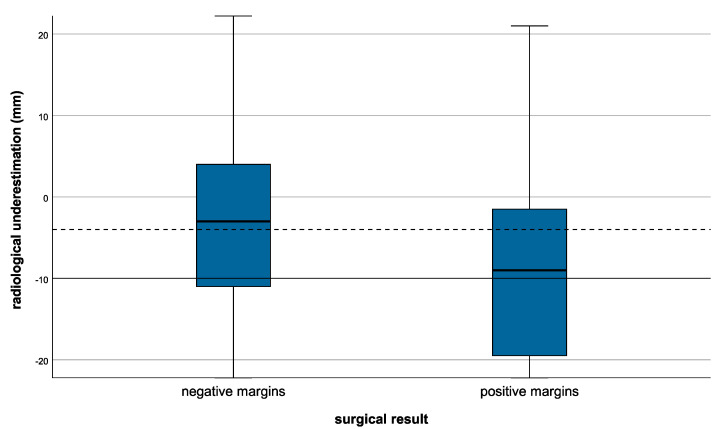
Radiological underestimation influencing the surgical result.

An underestimation of ≥10 mm was seen in 70 (37%) patients and an overestimation of ≥10 mm was seen in 26 (13.8%) patients, whereas 49.2% of the tumors were radiologically neither under- nor overestimated. The relationship between radiological underestimation and surgical results is demonstrated in Figure 1.

Mammographic underestimation of ≥10 mm was seen in 49.3% of patients with positive margins in contrast to 28.9% of patients with negative margins (*p* = 0.006; Table 1). The frequency of radiological underestimation differed significantly depending on the morphology of microcalcification. Of 70 patients with clinically relevant underestimation (≥10 mm), microcalcifications were recorded in 5 (7.1%) as linear, in 30 (42.9%) as fine pleomorphic, and in 35 (50.0%) cases as coarse heterogenous (*p* < 0.001). The distribution pattern of microcalcifications in underestimated DCIS was more frequently clustered in comparison with DCIS without relevant underestimation (71.4% vs. 37.0%; *p* < 0.001). Relevant underestimation was more frequent in mammographic tumor sizes ≤20 mm (85.7% vs. 44.5%; *p* < 0.001) (Table 3).

**Figure 2:**
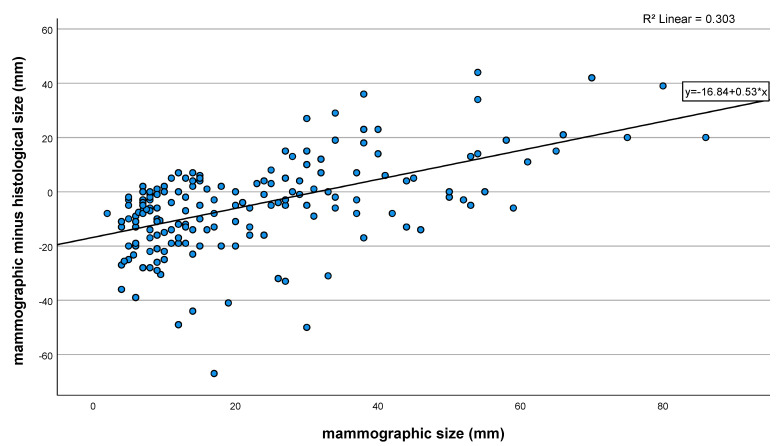
Relationship between mammographic size and radiological underestimation.

The scatter plot illustrates the relationship between radiological underestimation and mammographic tumor size. Relevant mammographic overestimation (>10 mm) of tumor size was observed in microcalcifications ≥30 mm (Figure 2).

There was a difference in the surgical extent of mammographic tumor sizes ≤20 mm vs. mammographic tumor sizes >20 mm. The median specimen size of mammographic tumor sizes ≤20 mm was 47 mm (range: 23–95 mm). In contrast, the median specimen size in mammographic tumor sizes >20 mm was 60 mm (range: 30–110). The difference was highly significant in the Mann–Whitney U test (*p* < 0.001).

Univariate regression revealed pleomorphic (including amorphous) microcalcifications (OR 3.77; 95%CI 1.27–11.18), clustered distribution patterns (OR 4.26; 95%CI 2.25–8.07), and mammographic tumor sizes ≤20 mm (OR 7.47; 95%CI 3.49–15.99) to be statistically significant associated with radiological underestimation ≥10 mm. Grading, estrogen receptor status, and comedo necrosis did not have a significant influence on radiological underestimation. After multivariable analysis, only a mammographic tumor size of ≤20 mm was identified as an independent risk factor (OR 6.49; 95%CI 2.30–18.26; *p* < 0.001; Table 4).

## 4. Discussion

Tumor size is the most limiting factor in reaching negative margins in breast-conserving surgery of pure DCIS. There is no doubt that margin status remains an important risk factor for local recurrence [29]. Positive margins depend on many factors; for example, comedo necrosis, radiological margins <10 mm [30,31], negative PR, tumor grade, and a larger DCIS size [20,31,32]. In the present study, the positive margin rate (PMR) after the initial surgery was 39.7%. This is in line with the results of systematic reviews, including seven studies with pure DCIS and a PMR ranging from 18 to 63% [15]. Our study has shown that a DCIS size of 25 mm or more was related to a sevenfold increased risk for positive margins. Mammographic size estimation does not always match with histological DCIS size. DCIS size is frequently underestimated by imaging. Recent studies reported mean differences of mammographic versus histological tumor sizes of 12.7 mm [33], 13 mm [34], and 16.5 mm [35].

Our results show a clinically relevant underestimation of ≥10 mm in 37% of patients. The mammographic underestimation of tumor size was an independent risk factor for positive margins in breast-conserving surgery of pure DCIS. These findings have been reported by other authors [15,31,36], but have never been further examined. Therefore, one aim of this retrospective study was to identify preoperatively known factors that are related to the mammographic underestimation of DCIS size. According to morphology and the distribution pattern of microcalcifications, we found that underestimation was significantly more frequent in pleomorphic microcalcifications in comparison to branched microcalcifications, and in clustered vs. ductal distribution patterns. To our knowledge, these results have not been described before. Other authors reported a correlation between underestimation and grading, meaning that high-grade DCIS is more often underestimated than low- and intermediate-grade DCIS [33]. The estrogen receptor <45% seems to be at the highest risk of underestimation independent from the DCIS size [36]. This cannot be supported by the current study.

We confirmed only mammographic sizes ≤20 mm to be independent risk factors of radiological underestimation ≥10 mm. This result is contrary to the results of Layfield et al. [33], which described that the discrepancy between mammographic and histological tumor size became greater with the increasing extent of mammographic DCIS size.

In a retrospective analysis of 34 patients with pure DCIS, radiological underestimation occurred significantly more often in histological DCIS sizes >2 cm [37]. In the current study, we correlate mammographic underestimation with preoperatively assessed mammographic size because the tumor size is not preoperatively known. Our results are meaningful because the planning of surgical management depends on radiological size estimation. Until now, there has been a lack of prospective studies that examine radiological underestimation and its determinants.

The extent of surgical resection was different in mammographic tumor sizes ≤20 mm. The median specimen size was 47 mm vs. 60 mm in mammographic tumor sizes >20 mm (*p* < 0.001). In anticipation of a mammographically smaller tumor, the surgical extent was less. However, in the knowledge that smaller tumors are more likely to be radiologically underestimated (as we show in our work), there should be an awareness among surgeons to remove a bigger specimen in order to achieve negative margins.

The routine use of preoperative magnetic resonance imaging (MRI) for the size estimation of DCIS is not recommended [2,4,15]. A recent meta-analysis revealed that preoperative MRIs did not have a significant impact on the surgical outcome or local recurrences [2,38]. However, a preoperative MRI increased the odds of having a mastectomy in the first surgery (adjusted OR 1.76, *p* 0.01) because MRI was more likely to detect a multicentric/multifocal DCIS [38]. Size correlation is more precise with an MRI compared to mammography [2]. Mammography underestimated high-grade DCIS by 10.5 mm compared to the MRI, by only 1 mm [39]. Therefore, patients with high-grade DCIS might be a subgroup that might benefit from further diagnostics [6]. Clustered calcifications seem to be an insufficient indicator in estimating tumor size [40]. Preoperative MRIs could reduce positive margins and re-excisions in patients with histologically proven DCIS without enhancing mastectomy rates [40,41,42]. The role of the preoperative MRIs in DCIS still remains unclear [15] and requires further investigation [4]. Another valuable imaging technique could be digital tomosynthesis, which demonstrated a small but significant benefit compared to mammograms regarding DCIS size estimation [35].

The evidence of cavity shave margins is not well approved. In literature research, there are not many studies concerning the benefit from cavity shave margins in ductal carcinoma in situ. Most studies draw attention to breast-conserving therapy of invasive breast cancer [43]. There are not as many results when searching for cavity shave margins in pure ductal carcinoma in situ. One trial [44] focused on that topic and showed a reduction in the positive margin rate. The influence of cavity shave margins in positive margins could be regarded further. However, evidence for routine use of these new techniques is low.

Our study has some limitations. Because of the pooled analysis, a part of the data is retrospective. Furthermore, there is a time gap between the two trials, so there might be no consistent study collective. A limitation might be that the mammograms and specimen radiographies were interpreted by only one breast radiologist (A.S.), but due to the high experience and the measurability of the lesion, this might not be very important. A strength is the large number of pure DCIS associated with microcalcifications included in this study. Moreover, due to the blind review of mammograms, a detailed analysis of several features of microcalcifications was possible. To our knowledge, this is the first study describing preoperatively known factors associated with the mammographic underestimation of DCIS size.

## 5. Conclusions

Radiological underestimation is an independent risk factor for positive margins in BCT of DCIS with microcalcifications. While planning and performing BCS, it must be considered that a relevant radiological underestimation is significantly more frequent in clustered DCIS with a mammographic size ≤20 mm.

## Figures and Tables

**Table 1:** Patient characteristics.

Variables	All Patients	Negative Margins	Positive Margins	*p* Value
	*n* = 189	*n* = 114	*n* = 75	
Age (years)				
*Mean (range)*	59.7 (34–84)	59.6 (35–84)	60 (34–81)	0.73
Specimen size (mm)				
*Median (range)*	50.0 (23–110)	52.5 (30–110)	48.0 (23–95)	0.009
Mammographic tumor size (mm)				
*Median (range)*	15 (2–86)	13 (2–70)	22 (2–86)	0.011
Histological tumor size (mm)				
*Median (range)*	25 (2–84)	17 (3–60)	31 (2–84)	<0.001
Grade of differentiation				0.36
*Low grade*	18 (9.5%)	13 (11.4%)	5 (6.7%)	
*Intermediate grade*	78 (41.3%)	49 (43%)	29 (38.7%)	
*High grade*	93 (49.2%)	52 (45.6%)	41 (54.7%)	
Estrogen receptor				0.039
*positive*	154 (84.2% *)	97 (89% *)	57 (77% *)	
*negative*	29 (15.8% *)	12 (11% *)	17 (23% *)	
Progesteron receptor				0.003
*positive*	120 (71% *)	77 (80.2% *)	43 (58.9% *)	
*negative*	49 (29% *)	19 (19.8% *)	30 (41.1% *)	
Radiological margins				0.004
*<5 mm*	111 (58.7%)	57 (50%)	54 (72%)	
*≥5 mm*	78 (41.3%)	57 (50%)	21 (28%)	
Radiological underestimation				0.006
*≥10 mm*	70 (37.0%)	33 (28.9%)	37 (49.3%)	
*<10 mm*	119 (63.0%)	81 (71.1%)	38 (50.7%)	
Intraoperative re-excision				0.524
*yes*	58 (30.7%)	33 (28.9%)	25 (33.3%)	
*no*	131 (69.3%)	81 (71.1%)	50 (66.7%)	

* Valid percentages (information on *n* = 5 missing).

**Table 2:** Factors associated with histologically positive margins on univariate and multivariable regression analysis among all patients undergoing BCS for DCIS with microcalcifications (*n* = 189).

Variable	Univariate Logistic Regression		Multivariable Logistic Regression	
	Odds Ratio (95% CI)	*p* Value	Odds Ratio (95% CI)	*p* Value
Specimen size				
≤50 mm vs. (vs.) >50 mm *	1.69 (0.94–3.05)	0.080	**2.51 (1.15–5.49)**	**0.021**
Mammographic tumor size				
>20 mm vs. ≤20 mm *	**2.05 (1.13–3.73)**	**0.018**	**5.46 (2.04–14.6)**	**0.001**
Histological tumor size **				
>25 mm vs. ≤25 mm *	**7.36 (3.82–14.2)**	**<0.001**		
Estrogen receptor				
Negative vs. positive *	**2.41 (1.07–5.41)**	**0.033**	0.75 (0.21–2.64)	0.659
Progesteron receptor				
Negative vs. positive *	**2.83 (1.42–5.61)**	**0.003**	2.13 (0.77–5.90)	0.145
Radiological margins				
<5 mm vs. ≥5 mm *	**2.57 (1.38–4.80)**	**0.003**	**2.71 (1.27–5.83)**	**0.010**
Mammographic underestimation				
≥10 mm vs. <10 mm *	**2.39 (1.30–4.39)**	**0.005**	**5.81 (2.39–14.12)**	**<0.001**

CI—confidence interval; * reference; ** was not included in multivariate regression model. Statistically significant Odds Ratio printed in bold

**Table 3:** Radiological underestimation of DCIS size dependent on multiple variables.

Variable	All Patients	No Relevant	Underestimation	*p* Value
	*n* = 189 (%)	Underestimation	≥10 mm	
		*n* = 119 (%)	*n* = 70 (%)	
Microcalcification				**0.013**
*Fine linear (branched)*	27 (14.3%)	22 (18.5%)	5 (7.14%)	
*Fine pleomorphic*	57 (30.2%)	28 (23.5%)	29 (41.4%)	
*Coarse heterogenous*	97 (51.3%)	62 (52.1%)	35 (50%)	
*amorphous*	8 (4.2%)	7 (5.9%)	1 (1.43%)	
Distribution pattern of microcalcification				**<0.001**
*Ductal/segmental*	95 (50.3%)	75 (63.0%)	20 (28.6%)	
*clustered*	94 (49.7%)	44 (37.0%)	50 (71.4%)	
Comedo necrosis				0.251
*yes*	153 (81%)	93 (78.2%)	60 (85.7%)	
*no*	36 (19%)	26 (21.8%)	10 (14.3%)	
Grading				0.689
*Low grade*	18 (9.5%)	13 (10.9%)	5 (7.1%)	
*Intermediate grade*	78 (41.3%)	48 (42.9%)	30 (42.9%)	
*Hgh grade*	93 (49.2%)	58 (48.7%)	35 (50%)	
Estrogen receptor				0.402
*positive*	154 (84.2% *)	97 (85.1% *)	57 (82.6% *)	
*negative*	29 (15.8% *)	17 (14.9% *)	12 (17.4% *)	
Progesteron receptor				0.861
*Positive*	120 (71% *)	76 (71.7% *)	44 (69.8% *)	
*negative*	49 (29% *)	30 (28.3% *)	19 (30.2% *)	
Mammographic tumor size				**<0.001**
*≤20 mm*	113 (59.8%)	53 (44.5%)	60 (85.7%)	
*>20 mm*	76 (40.2%)	66 (55.5%)	10 (14.3%)	

* Valid percentages (information on *n* = 5 missing); Statistically significant Odds Ratio printed in bold.

**Table 4:** Preoperative known parameter of radiological underestimation ≥10 mm.

Variable	Univariate Logistic Regression		Multivariable Logistic Regression	
	Odds Ratio (95% CI)	*p* Value	Odds Ratio (95% CI)	*p* Value
**Microcalcification** Fine linear (branched) * ***Fine pleomorphic*** ***heterogeneous***	**3.77 (1.27–11.18)** 2.48 (0.86–7.14)	**0.017** 0.091	2.87 (0.85–9.69) 1.75 (0.54–5.72)	0.163 0.089 0.354
**Distribution pattern of Microcalcification** Ductal * vs. *clustered clustered*	**4.26 (2.25–8.07)**	**<0.001**	0.87 (0.34–2.22)	0.764
**Comedo necrosis** no * vs. *yes*	1.68 (0.76–3.73)	0.204		
**Grading** G1 * vs. *G2 G3*	1.63 (0.53–5.02) 1.57 (0.52–4.78)	0.399 0.428		
**Estrogenreceptor** positive * vs. *negative negative*	1.20 (0.54–2.69)	0.657		
**Mammographic DCIS Size** > 20 mm * vs. *** ≤ 20 mm***	**7.47 (3.49–15.99)**	**<0.001**	**6.49 (2.30–18.26)**	**<0.001**

* Marks the reference category.

## Data Availability

The data presented in this study are available upon request from the corresponding author.
